# The role of mitomycin in the treatment of non-small cell lung cancer: a systematic review with meta-analysis of the literature

**DOI:** 10.1054/bjoc.2001.1742

**Published:** 2001-05

**Authors:** J P Sculier, L Ghisdal, T Berghmans, F Branle, J J Lafitte, F Vallot, A P Meert, F Lemaitre, E Steels, A Burniat, C Mascaux, M Paesmans

**Keywords:** mitomycin, non-small cell lung cancer, meta-analysis, systematic review, chemotherapy

## Abstract

In order to clarify the role of mitomycin (MMC) in the treatment of NSCLC, we performed a systematic review of the literature and qualitatively assessed the selected studies using the ELCWP and Chalmers scales. 5 trials (202 patients) assessed the activity of MMC as single-agent chemotherapy in NSCLC. The overall response rate was 25% (95% Cl 19–31). In 10 randomized phase III trials (1769 patients), we studied the role of MMC in combination therapy. A meta-analysis, based on the available published data, failed to show any survival advantage of the MMC containing regimens (hazard ratio = 0.95; 95% Cl 0.83–1.10). Finally, 4 eligible trials (139 patients) assessed the activity of MMC regimens as salvage therapy, 3 in combination with vindesine and one with cisplatin and vinblastine. The overall response rate for the MMC-vindesine regimen was 10.5% (95% Cl 1.7–19.4). In conclusion, MMC is an active drug for NSCLC but does not improve survival when combined with other active drugs, particularly cisplatin. Its use for salvage therapy appears to be associated with marginal activity only. © 2001 Cancer Research Campaign http://www.bjcancer.com


*British Journal of Cancer* (2001) **84**(9), 1150–1155
© 2001 Cancer Research Campaign
doi: 10.1054/ bjoc.2001.1742, available online at http://www.idealibrary.com on IDEAL®

http://www.bjcancer.com

### The role of mitomycin in the treatment of non-small cell lung cancer: a systematic review with meta-analysis of the literature

**JP Sculier, L Ghisdal, T Berghmans, F Branle, JJ Lafitte, F Vallot, AP Meert, F Lemaitre, E Steels, A Burniat, C Mascaux and M Paesmans for the European Lung Cancer Working Party (ELCWP)**

**Summary** In order to clarify the role of mitomycin (MMC) in the treatment of NSCLC, we performed a systematic review of the literature and qualitatively assessed the selected studies using the ELCWP and Chalmers scales. 5 trials (202 patients) assessed the activity of MMC as single-agent chemotherapy in NSCLC. The overall response rate was 25% (95% Cl 19–31). In 10 randomized phase III trials (1769 patients), we studied the role of MMC in combination therapy. A meta-analysis, based on the available published data, failed to show any survival advantage of the MMC containing regimens (hazard ratio = 0.95; 95% Cl 0.83–1.10). Finally, 4 eligible trials (139 patients) assessed the activity of MMC regimens as salvage therapy, 3 in combination with vindesine and one with cisplatin and vinblastine. The overall response rate for the MMC-vindesine regimen was 10.5% (95% Cl 1.7–19.4). In conclusion, MMC is an active drug for NSCLC but does not improve survival when combined with other active drugs, particularly cisplatin. Its use for salvage therapy appears to be associated with marginal activity only. © 2001 Cancer Research Campaign http://www.bjcancer.com

**Keywords**: mitomycin; non-small cell lung cancer; meta-analysis; systematic review; chemotherapy

In the 1990s, chemotherapy has been shown able to improve survival of patients presenting with advanced non-small cell lung cancer (NSCLC, 1995). The survival benefit was obtained with first-generation active cytostatic agents – mainly ifosfamide, vinblastine, vindesine, mitomycin (MMC) – in combination with cisplatin (Donnadieu et al, 1991). New active drugs – the second generation – have appeared during the last decade, including gemcitabine, paclitaxel, docetaxel, vinorelbine and irinotecan. Their role in addition or in place of the first-generation agents, which should not be considered as obsolete for the unique reason that they are older and less fashionable, has yet to be defined (Meert et al, 1999).

In this context, we have performed a systemic review of the literature about the role of one of the first-generation drugs, mitomycin (MMC), in the management of NSCLC. In order to determine if it is worthy to further conduct trials with that agent, we have searched answers to the 3 following questions: (1) is MMC an active drug against NSCLC? (2) does MMC improve the results when added to other active agents? (3) is MMC useful for salvage therapy? We have performed this investigation by using a methodology similar to that we have already used to conduct evidence-based medicine analyses of the literature.

### MATERIAL AND METHODS

To be eligible for the systematic review, a trial had to fulfil the following criteria: to deal only with NSCLC, to have been published as a full paper in the English or French language, to have a prospective design and to assess the effect of MMC in a randomized trial or in a first-line or second-line phase II trial according to the studied question.

Trials were identified by an electronic search (Medline) in addition to the use of the personal bibliography of one of the authors and by consulting the references reported in the selected articles.

Each trial was read and assessed for methodology by 12 investigators, including 11 physicians and 1 biostatistician. Each investigator independently extracted the data from the articles and disagreements were resolved by consensus. Randomized trials were evaluated for methodology by 2 quality scores calculated on the basis of the data reported in the publications: the score proposed by Chalmers et al (1981) and used by Marino in two meta-analyses (Marino et al, 1994, 1995); and the score proposed by the ELCWP (European Lung Cancer Working Party) (Mascaux et al, 2000). Phase II trials were assessed by the ELCWP score for phase II studies (Meert et al, 1999).

The result of a phase III trial was considered as conclusive if the *P* value for the statistical test comparing the survival distributions between arms for the overall patients populations was <0.05 in favour of the experimental arm. The trial was then called ‘positive’. In the other situations (statistically significant survival benefit for the control arm or non-statistically significant difference in survival distributions), it was called ‘negative’.

The association between the quality scores or between a quality score treated as continuous variable and another continuous variable was measured by the Spearman ranks correlation coefficient. Its significance was assessed by testing a null hypothesis of equality to zero of this coefficient. The comparison between quality scores according to the value of a discrete variable was made by non-parametric Mann–Whitney (for dichotomic variables) or Kruskal–Wallis (for nominal variables with multiple classes) tests. To compare regimens according to the drug dose-intensity, we used the theoretical dose-intensity for the drug

*Received 22 August 2000*
*Revised 6 December 2000*
*Accepted 22 January 2001*

*Correspondence to:* JP Sculier

(reported in mg/m²/week) by dividing the theoretical absolute dose administered by the theoretical duration of the treatment before response evaluation (if not reported, we made the calculations with 3 cycles of treatment).

Confidence intervals (CI) for the response rate to the chemotherapy regimen were, for consistency, recalculated using the exact binomial distribution.

For objective response, the numbers of eligible and assessable patients were recorded in each article as described by the authors.

For the quantitative aggregation of the antitumoral response results and the reported toxic deaths, we measured the treatment effects using the odds ratios calculated on the contingency tables observed in each trials. The individual odds ratios were combined using the Peto method after having tested the homogeneity of the odds ratios estimated in each study.

For the quantitative aggregation of the survival results reported in the randomised phase III trials, we measured the treatment effect by the hazard ratio (HR) between the survival distributions. For each trial, this HR was estimated by a method depending on the results provided in the publications. The most accurate method consisted to calculate the estimated HR and its standard error using 2 of the following parameters: the HR point estimate, the log-rank statistic or its *P* value, the O-E statistic (difference between numbers of observed and expected events) or its variance. If not available, we looked for the total number of events and the log-rank statistic or its *P* value allowing calculation of an approximation of the HR estimate. Finally, if it was impossible to apply the second method, we extracted from the graphical representation of the survival distributions survival rates at some specified times chosen on a trial by trial basis in order to reconstruct the log-rank statistic and its variance. The individual HR point estimates were combined after acceptation of the null hypothesis of the homogeneity of the treatment effect across the various trials, using the Peto method in order to obtain a global HR estimate of the treatment effect. By convention, a HR < 1 implied a survival benefit for the experimental arm.

All reported *P* values are two-tailed.

### RESULTS

A total of 19 trials were found to be eligible for the present systematic review: 5 phase II trials assessing MMC as first-line single-agent therapy, 10 phase III randomized trials assessing the addition of MMC to a basic active regimen and 4 phase II trials assessing the role of MMC-containing regimens as second-line chemotherapy for advanced NSCLC.

### Question 1: is MMC an active drug against NSCLC?

5 studies (Israel et al, 1975; Samson et al, 1978; Ruckdeschel et al, 1981; Niell et al, 1989; Veeder et al, 1992a) including 202 assessable patients provided results about the activity of MMC when given as first-line single-agent therapy. Their design is phase II for 3 and randomized phase III for 2. 3 specifically deal with squamous cell carcinoma and one with adenocarcinoma and large cell carcinoma. Their main characteristics and results are shown in Table 1.

The global quality score ranged between 26.1% and 81.9% with a median of 57.3%. There was a good correlation between the score and the year of publication (r = 1) while no significant correlation was found with the number of eligible patients (r = 0.6; *P* = 0.55).

The reported response rates ranged from 19 to 50%, with an overall response rate of 25% (95% Cl: 19–32%).

### Question 2: does MMC improve the results when added to other active agents?

10 randomized trials comparing a basic chemotherapy regimen with or without MMC (Einhorn et al, 1986; Crino et al, 1988, 1990; Bonomi et al, 1989, Luedke et al, 1990; Fukuoka et al, 1991; Shinkai et al, 1991; Weick et al, 1991; Mylonakis et al, 1992; Gandara et al, 1993; Masutani et al, 1996) were available for a meta-analysis. Their main characteristics and results are summarized in Table 2 8 of them included more than 2 arms but we considered only the 2 arms of interest, leading to a total of 1769 eligible patients, 876 treated in the experimental arm with MMC and 893 in the control arm without MMC. As shown in Table 2, most often the data were missing to consider the number of randomized patients for the aggregation. The basic chemotherapy regimen was cisplatin–vindesine in 4 trials, cisplatin–vinblastine in 3 and cisplatin–etoposide, cisplatin or vindesine in 1 each. In 7 trials, a reduction of the dosage of at least one of the basic drugs was performed in the MMC arm. Significant improvement of response rate was reported in 3 trials but none was associated with significant survival improvement. All the trials were thus considered as negative.

The quality scores of the randomized trials are reported in Table 3. The ELCWP score ranged from 52.8% to 86.8% with a median of 66.5% and the Chalmers one from 30.6% to 64.8% with a median of 43.5%. There was a significant correlation between both scales (r = 0.68; *P* = 0.03). We found no significant correlation between the scores and the number of eligible patients included into the study, the date of study activation or the date of publication.

**Table 1** Characteristics and results of the studies assessing the role of MMC as first-line single-agent therapy

| Reference | *n* assessable patients | MMC DI (mg/m²/wk) | OR (%) 95% CI (%) | ELCWP IV (%) | ELCWP EV (%) | ELCWP QS (%) |
|---|---|---|---|---|---|---|
| Israel et al, 1975 | 20 | 0.02 mg/kg | 10 (50%) 26% – 74% | 22.2 | 28.9 | 26.1 |
| Samson et al, 1978 | 37 | 2.78 | 7 (20%) 5% – 33% | 50 | 36.5 | 42 |
| Ruckdeschel et al, 1981 | 28 | 6.67 | 5 (19%) 2% – 34% | 50 | 61.7 | 57.3 |
| Niell et al, 1989 | 53 | 2.5 | 10 (19%) 7% – 30% | 69.4 | 70.7 | 70.2 |
| Veeder et al, 1992b | 64 | 2.78 | 19 (30%) 18% – 42% | 83.3 | 81 | 81.9 |
| Overall | 202 | | 51 (25.2%) 19% – 32% | | | |

MMC: mitomycin C, DI: dose-intensity (mg/m²/wk), OR: objective response; IV: internal validity; EV: external validity; QS: quality score.

© 2001 Cancer Research Campaign 

**Table 2** Characteristics and reported results of the randomized studies assessing the role of the addition of MMC to a basic regimen

| Reference | *n* eligible patients (*n* randomized) | | Basic chemotherapy regimen | OR rate (%) | | | MS time (wks) | | |
|---|---|---|---|---|---|---|---|---|---|
| | MMC arm | Control arm | | MMC arm | Control arm | *P* | MMC arm | Control arm | *P* |
| Einhorn et al, 1986 | 41 (?) | 41 (?) | CDDP-VDS* | 20 | 27 | NS | 17 | 26 | NS |
| Crino et al, 1990 | 57 (?) | 69 (?) | CDDP-VP16 | 26 | 30 | NS | 37 | 35 | NS |
| Bonomi et al, 1989 | 176 (?) | 175 (?) | CDDP-VBL* | 20 | 13 | S | 23 | 25 | NS |
| Luedke et al, 1990 | 143 (?) | 141 (?) | VDS* | 27 | <1 | S | 20 | 15 | NS |
| Fukuoka et al, 1991 | 68 (69) | 67 (68) | CDDP-VDS* | 43 | 33 | NS | 42 | 50 | NS |
| Shinkai et al, 1991 | 61 (62) | 63 (64) | CDDP-VDS* | 35 | 23 | NS | 45 | 39 | NS |
| Weick et al, 1991 | 134 (139) | 142 (156) | CDDP-VBL* | 24 | 17 | NS | 25 | 21 | NS |
| Mylonakis et al, 1992 | 51 (51) | 52 (52) | CDDP-VBL | 18 | 31 | NS | 32 | 35 | NS |
| Gandam et al, 1993 | 110 (?) | 108 (?) | CDDP | 27 | 14 | S | 54 | 40 | NS |
| Masutani et al, 1996 | 35 (?) | 35 (?) | CDDP-VDS* | 43 | 29 | NS | 33 | 36 | NS |

OR: objective response; MS: median survival; S: significant; NS: non significant; CDDP: cisplatin; VDS: vindesine; VBL: vinblastine; * = with reduction of the dosage of the basic drugs in the MMC-experimental arm.

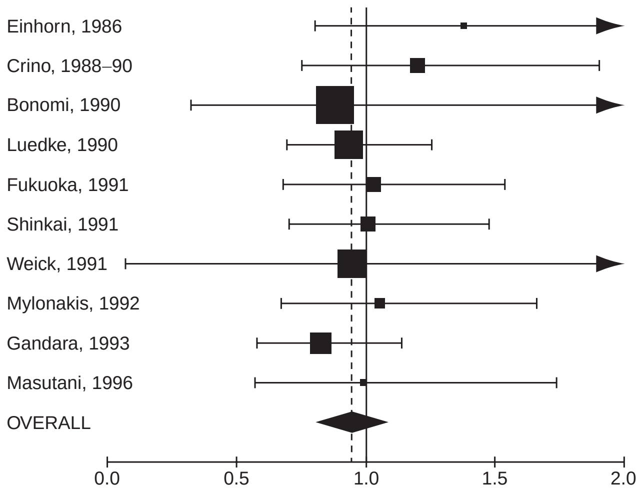

**Figure 1** Results of the survival meta-analysis of the 10 randomized trials comparing a chemotherapy regimen with or without MMC

No significant bias being found in quality scores between the trials, a meta-analysis of survival and response results was performed.

For survival, the individual HR were calculated by one of the methods reported in the Material and methods section. In one case, the HR was estimated using the total number of events and the log-rank statistic, in 7 using extracted survival rates from the graphical representations of the survival distributions and in 2 using the global number of events in each arm. We aggregated firstly all the trials and secondly a subgroup of the 3 trials that respected the same drug dosage in the 2 arms. These meta-analyses failed to show a significant difference between the regimens with or without MMC. The individual and pooled hazard ratios (HR) are shown in Figures 1 and 2. The aggregation of the 10 randomized trials resulted in an overall HR of 0.95 (95% confidence interval or CI: 0.83–1.10) while the pooled HR for the 3 trials subgroup was 1.05 (95% CI: 0.83–1.33). The test for heterogeneity of the treatment effect was not significant ($P = 0.93$).

For response, the analysis had to deal with heterogeneity of the effect of the addition of MMC mainly due to the study of Luedke et al (1990) who compared vindesine with or without MMC.

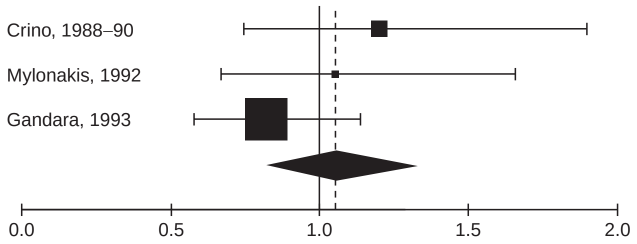

**Figure 2** Results of the survival meta-analysis of the subgroup of 3 trials comparing a chemotherapy regimen with or without MMC, without drug dosage reduction

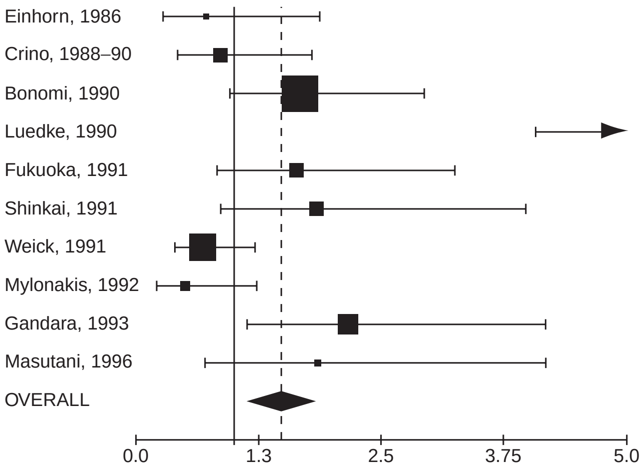

**Figure 3** Results of the objective response rate meta-analysis of the 10 randomized trials comparing a chemotherapy regimen with or without MMC

As shown in Figures 3 and 4, when this study is included, a significant odds-ratio (OR) was obtained in favour of MMC-containing regimens: 1.48 (95% CI 1.17–1.86); when it is excluded, the OR (test for heterogeneity: $P = 0.54$) is not anymore significantly associated with an improved response rate: 1.21 (95% CI 0.95–1.54).

The number of observed toxic deaths was not significantly different between the arms with or without MMC, with an OR of 1.55 (95% CI: 0.73–3.28; $P = 0.98$; test for heterogeneity: $P = 0.53$).

 *© 2001 Cancer Research Campaign*

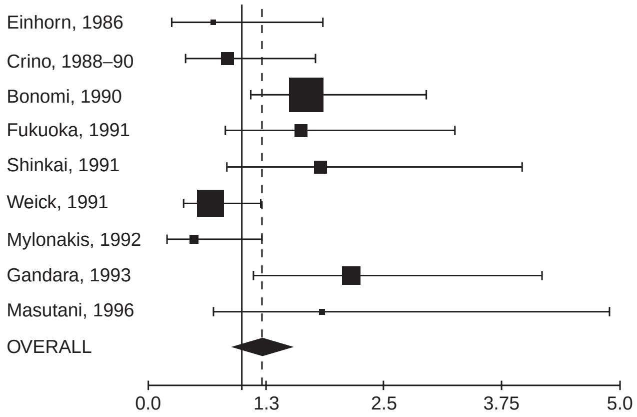

**Figure 4** Results of the objective response rate meta-analysis of the randomized trials after exclusion of the study by Luedke et al (1990)

### Question 3: is MMC useful for salvage therapy?

On the 4 prospective phase II trials found in the literature (Table 4), 3 deal with a combination of MMC with vindesine (Kris et al, 1985; Sculier et al, 1986, Bonomi et al, 1989) and 1 with cisplatin and vinblastine (Gridelli et al, 1992). The ELCWP quality score for phase II trials ranged from 21.1% to 75% with a median of 61.1%. The response rate ranged for the MMC–vindesine regimen from 0% to 17%, with an overall response rate of 10.5% (95% CI: 1.7–19.4%). It was 6% for the cisplatin–vinblastine–MMC combination.

### DISCUSSION

Our systematic review intended to answer 3 questions about the role of mitomycin in the management of advanced NSCLC. A response to 2 of them is possible: MMC is an active drug for this disease as shown by the phase II trials testing its activity as single-agent first-line chemotherapy and the meta-analysis of the randomized trials fails to obtain any survival advantage when MMC is added to a basic combination regimen with first-generation cytostatic agents (mainly cisplatin and/or vinca alcaloids). For the last question concerning the role of the drug for salvage chemotherapy, the response is less evident because of the limited number of published studies on the topic.

To perform our systematic review, we have used a methodology that was similar to prior studies of this type reported by our Group. The principle is to assess the trial quality by methodological scales – the Chalmers and ELCWP scores – in order to search for potential methodological aspects of the published trials that might explain heterogeneity of the reported treatment effects. If there is no significant difference among the publications, as in the present report, we go further in our analysis and perform a quantitative aggregation (meta-analysis) of the results of the individual trials. This approach applied to MMC in advanced NSCLC was relatively easy because all the trials provide similar results. All the phase II studies assessing the role of MMC as first-line single-agent therapy revealed that the drug is active and none of the randomized studies assessing the role of the addition of MMC to a basic regimen showed a survival advantage for the experimental arm. We had thus not to compare for quality 'positive' and 'negative' studies. The only significant finding was an improved quality in favour of more recently published phase II trials, which can be very well explained by the amelioration of the trials methodology obtained over the 3 last decades.

Mitomycin is associated with a 25% objective response rate when administered as single-agent first-line chemotherapy in advanced NSCLC. Most of the authors consider that the cut-off to consider a drug as active in this disease is defined by the observation of response rates above 15 to 20%. The results reported by the 5 trials described in Table 1 are consistent, despite being conducted over a relatively long period (~25 years). Some of them were performed with some specific histological subtypes of NSCLC but we do not believe that this heterogeneity is a potential source of bias. The meta-analysis that we performed about response in NSCLC failed to show significantly different effects according to the histological type.

The next step of the systematic review was to identify the potential benefit for the patient of the inclusion of MMC in the chemotherapy treatment. The primary endpoint that we choose was survival, the endpoint usually used by the investigators for phase III randomized trials. Other endpoints that could be of interest for the patients are symptom control, quality of life or toxicity of the treatment but the publications reported no data or too poorly described data to allow a meaningful aggregation. The main problem in the interpretation of the results of our meta-analysis is, in 7 of the 10 randomized trials, a dosage reduction of the non-MMC drugs in the experimental arm compared to control arm. For this reason, as shown in Figures 1 and 2, we performed two meta-analyses, one with all the studies and another with the 3 studies with the purest design to address our question. None revealed a survival advantage for MMC-regimens but due to the small number of adequately designed randomized trials

**Table 3** Quality scores of the randomized studies assessing the role of the addition of MMC to a basic regimen

| Reference | Chalmers score % | | | ELCWP score % | | |
|---|---|---|---|---|---|---|
| | Internal validity | External validity | Total | Protocol design | Performance analysis | Total |
| Einhorn, 1986 | 50 | 18 | 40.9 | 61.9 | 50 | 54.8 |
| Crino, 1988/90 | 28.6 | 36 | 30.6 | 66.7 | 66.1 | 66.4 |
| Bonomi, 1990 | 38.1 | 12 | 30.6 | 50 | 54.8 | 52.9 |
| Luedke, 1990 | 38.1 | 30 | 35.8 | 52.4 | 53.1 | 52.8 |
| Fukuoka, 1991 | 57.1 | 48 | 54.6 | 90.5 | 71.9 | 79.3 |
| Shinkai, 1991 | 71.4 | 48 | 64.8 | 85.7 | 87.5 | 86.8 |
| Weick, 1991 | 47.6 | 42 | 46 | 61.9 | 73.4 | 68.9 |
| Mylonakis, 1992 | 47.6 | 48 | 47.7 | 73.8 | 53.1 | 61.3 |
| Gandara, 1993 | 47.6 | 48 | 47.7 | 59.5 | 71.7 | 66.7 |
| Masutani, 1996 | 38.1 | 42 | 39.2 | 61.9 | 74.2 | 69.2 |

*© 2001 Cancer Research Campaign* 

**Table 4** Characteristics and results of the studies assessing the role of MMC-containing regimens as second-line chemotherapy

| Reference | *n* assessable patients | Other drugs of the regimen | MMC DI | OR (%) 95% CI | ELCWP IV (%) | ELCWP EV (%) | ELCWP QS (%) |
|---|---|---|---|---|---|---|---|
| Kris et al, 1985 | 29 | VDS | 2.5 | 5(17%) 2–33% | 72.2 | 57.7 | 63.6 |
| Sculier et al, 1986 | 16 | VDS | 2.5 | 1 (6%) 0–21% | 75 | 75 | 75 |
| Bonomi et al, 1989 | 88 | CDDP + VBL | 3.3 | 5(6%) 0–11% | 22.2 | 20.4 | 21.1 |
| Gridelli et al, 1992 | 12 | VDS | 2.5 | 0 | 44.4 | 63.7 | 58.5 |

MMC: mitomycin C, DI: dose-intensity (mg/m$^2$/wk), OR: objective response; IV: internal validity; EV: external validity; QS: quality score; VDS: vindesine; CDDP: cisplatin; VBL: vinblastine.

(only 3), the provided evidence might not be sufficient to change practice.

Although of less interest, we aggregated also the response rates. We had to deal in the analysis with a heterogeneity problem because of the trial of Luedke et al (1990) who reported in a very large trial a very significantly improved response rate when MMC was added to vindesine but without significant effect on survival. This trial may be criticized because the response rate obtained for vindesine was very low and is thus not in agreement with the majority of the other studies published with this drug in the literature. When this trial is omitted for meta-analysis (Figure 4), there is no response rate improvement by the addition of MMC while when it is included (Figure 3), there is a positive advantage in favour of MMC-containing chemotherapy.

The last point that we analysed was the role of MMC in salvage chemotherapy regimens (Table 4). In fact, the only regimen that has been the topic of publications is MMC–vindesine. The response rate reported is around 10%, which is rather marginal. In fact, we believe that the available literature for this question has to be considered as non-conclusive because of a lack of sufficient data, including about first-line chemotherapy characteristics.

In conclusion, the present systematic review shows that MMC is an active drug against advanced NSCLC but does not improve survival when added to other first-generation active cytostatic agents like cisplatin, vindesine and vinblastine. It should not be anymore used in this indication. Nevertheless its role for salvage chemotherapy or in combination with the second-generation active drugs require to be studied in further investigations.

### REFERENCES

Bonomi PD, Finkelstein DM, Ruckdeschel JC, Blum RH, Green MD, Mason B, Hahn R, Tormey DC, Harris J and Comis R (1989) Combination chemotherapy versus single agents followed by combination chemotherapy in stage IV non-small-cell lung cancer: a study of the Eastern Cooperative Oncology Group. *J Clin Oncol* **7**: 1602–1613

Chalmers TC, Smith H Jr, Blackburn B, Silverman B, Schroeder B, Reitman D and Ambroz A (1981) A method for assessing the quality of a randomized control trial. *Control Clin Trials* **2**: 31–49

Crino L, Darwish S, Corgna E, Meacci ML, Di Costanzo F, Buzzi F, Fornari G, Santi F, Ballatori E and Luccioli L (1988) Treatment of advanced non-small cell lung cancer (NSCLC): the “Umbria” cooperative study. *Semin Oncol* **15**: 52–55

Crino L, Tonato M, Darwish S, Meacci ML, Corgna E, Di Costanzo F, Buzzi F, Fornari G, Santi E and Ballatori E (1990) A randomized trial fo three cisplatin-containing regimens in advanced non-small-cell lung cancer (NSCLC): a study of the Umbrian Lung Cancer Group. *Cancer Chemother Pharmacol* **26**: 52–56

Donnadieu N, Paesmans M and Sculier JP (1991) Chimiothérapie du cancer bronchique non à petites cellules: méta-analyse de la littérature en fonction de l’extension de la maladie. *Rev Mal Respir* **8**: 197–204

Einhorn LH, Loehrer PJ, Williams SD, Meyers S, Gabrys T, Nattan SR, Woodburn R, Drasga R, Songer J and Fisher W (1986) Random prospective study of vindesine versus vindesine plus high-dose cisplatin versus vindesine plus cisplatin plus mitomycin C in advanced non-small-cell lung cancer. *J Clin Oncol* **4**: 1037–1043

Fukuoka M, Masuda N, Furuse K, Negoro S, Takada M, Matsui K, Takifuji N, Kudoh S, Kawahara M and Ogawara M (1991) A randomized trial in inoperable non-small-cell lung cancer: vindesine and cisplatin versus mitomycin, vindesine, and cisplatin versus etoposide and cisplatin alternating with vindesine and mitomycin. *J Clin Oncol* **9**: 606–613

Gandara DR, Crowley J, Livingston RB, Perez EA, Taylor CW, Weiss G, Neefe JR, Hutchins LF, Roach RW and Grunberg SM (1993) Evaluation of cisplatin intensity in metastatic non-small-cell lung cancer: a phase III study of the Southwest Oncology Group. *J Clin Oncol* **11**: 873–878

Gridelli C, Airoma G, Incoronato P, Pepe R, Palazzolo G, Rossi A and Bianco AR (1992) Mitomycin C plus vindesine or cisplatin plus epirubicin in previously treated patients with symptomatic advanced non-small-cell lung cancer. *Cancer Chemother Pharmacol* **30**: 212–214

Israel L, Chahinian P and Depierre A (1975) Response of 65 measurable epidermoid bronchogenic tumors of known spontaneous doubling time to four different chemotherapeutic regimens – strategic deductions. *Medical and Pediatrics Oncology* **1**: 83–93

Kris MG, Gralla RJ, Kelsen DP, Casper ES, Burke MT, Fiore JJ, Cibas IR and Heelan RT (1985) Trial of vindesine plus mitomycin in stage-3 non-small cell lung cancer. An active regimen for outpatient treatment. *Chest* **87**: 368–372

Luedke DW, Einhorn L, Omura GA, Sarma PR, Bartolucci AA, Birch R and Greco FA (1990) Randomized comparison of two combination regimens versus minimal chemotherapy in nonsmall-cell lung cancer: a Southeastern Cancer Study Group Trial. *J Clin Oncol* **8**: 886–891

Marino P, Pampallona S, Preatoni A, Cantoni A and Invernizzi F (1994) Chemotherapy vs supportive care in advanced non-small-cell lung cancer. Results of a meta-analysis of the literature. *Chest* **106**: 861–865

Marino P, Preatoni A, Cantoni A and Buccheri G (1995) Single-agent chemotherapy versus combination chemotherapy in advanced non-small cell lung cancer: a quality and meta-analysis study. *Lung Cancer* **13**: 1–12

Mascaux C, Paesmans M, Berghmans T, Branle F, Lafitte JJ, Lemaitre F, Meert AP, Vermylen P and Sculier JP (2000) A systematic review of the role of etoposide and cisplatin in the chemotherapy of small cell lung cancer with methodology assessment and meta-analysis. *Lung Cancer* **30**: 23–36

Masutani M, Akusawa H, Kadota A, Ohchi Y, Takahashi N, Tanigawa S, Koya Y and Horie T (1996) A phase III randomized trial of cisplatin plus vindesine versus cisplatin plus vindesine plus mitomycin C versus cisplatin plus vindesine plus ifosfamide for advanced non-small-cell lung cancer. *Respirology* **1**: 49–54

Meert AP, Berghamans T, Branle F, Lemaitre F, Mascaux C, Rubesova E, Vermylen P, Paesmans M and Sculier JP (1999) Phase II and III studies with new drugs for non-small cell lung cancer: a systematic review of the literature with a methodology quality assessment. *Anticancer Res* **19**: 4379–4390

Mylonakis N, Tsavaris N, Bacoyiannis C, Karvounis N, Kakolyris S, Karabelis A, Beer M and Kosmidis P (1992) A randomized prospective study of cisplatin and vinblastine versus cisplatin, vinblastine and mitomycin in advanced non-small cell lung cancer. *Ann Oncol* **3**: 127–130

Niell HB, Griffin JP, Hunter RF, Meredith CA and Somes G (1989) Combination versus sequential single-agent chemotherapy in the treatment of patients with advanced non-small cell lung cancer. *Med Pediatr Oncol* **17**: 69–75

Non-small Cell Lung Cancer Collaborative Group (1995) Chemotherapy in non-small cell lung cancer: a meta-analysis using updated data on individual patients from 52 randomised clinical trials. *BMJ* **311**: 899–909

Ruckdeschel JC, Mehta CR, Salazar OM, Cohen M, Vogl S, Koons LS and Lerner H (1981) Chemotherapy for inoperable, non-small cell bronchogenic carcinoma: EST 2575, generation II. *Cancer Treat Rep* **65**: 965–972

 *© 2001 Cancer Research Campaign*

Samson MK, Comis RL, Baker LH, Ginsberg S, Fraile RJ and Crooke ST (1978) Mitomycin C in advanced adenocarcinoma and large cell carcinoma of the lung. *Cancer Treat Rep* **62**: 163–165

Sculier JP, Klastersky J, Dumont JP, Vandermoten G, Rocmans P, Libert P, Ravez P, Becquart D, Mommen P and Dalesio O (1986) Combination chemotherapy with mitomycin and vindesine in advanced non-small cell lung cancer: a pilot study by the Lung Cancer Working Party (Belgium). *Cancer Treat Rep* **70**: 773–775

Shinkai T, Eguchi K, Sasaki Y, Tamura T, Ohe Y, Kojima A, Oshita F and Saijo N (1991) A randomised clinical trial of vindesine plus cisplatin versus mitomycin plus vindesine and cisplatin in advanced non-small cell lung cancer. *Eur J Cancer* **27**: 571–575

Veeder MH, Jett JR, Su JQ, Mailliard JA, Foley JF, Dalton RJ, Etzell PS, Marschke RFJ, Kardinal CG and Maksymiuk AW (1992a) A phase III trial of mitomycin C alone versus mitomycin C, vinblastine, and cisplatin for metastatic squamous cell lung carcinoma. *Cancer* **70**: 2281–2287

Veeder MH, Jett JR, Su JQ, Mailliard JA, Foley JF, Dalton RJ, Etzell PS, Marschke RF, Jr., Kardinal CG and Maksymiuk AW (1992b) A phase III trial of mitomycin C alone versus mitomycin C, vinblastine, and cisplatin for metastatic squamous cell lung carcinoma. *Cancer* **70**: 2281–2287

Weick JK, Crowley J, Natale RB, Hom BL, Rivkin S, Coltman CAJ, Taylor SA and Livingston RB (1991) A randomized trial of five cisplatin-containing treatments in patients with metastatic non-small-cell lung cancer: a Southwest Oncology Group study. *J Clin Oncol* **9**: 1157–1162

*© 2001 Cancer Research Campaign*

## References

[PDF_00401] Bonomi P. D., Finkelstein D. M., Ruckdeschel J. C., Blum R. H., Green M. D., Mason B., Hahn R., Tormey D. C., Harris J., Comis R. (1989). Combination chemotherapy versus single agents followed by combination chemotherapy in stage IV non-small-cell lung cancer: a study of the Eastern Cooperative Oncology Group.. J Clin Oncol.

[PDF_00406] Chalmers T. C., Smith H., Blackburn B., Silverman B., Schroeder B., Reitman D., Ambroz A. (1981). A method for assessing the quality of a randomized control trial.. Control Clin Trials.

[PDF_00412] Crino L., Tonato M., Darwish S., Meacci M. L., Corgna E., Di Costanzo F., Buzzi F., Fornari G., Santi E., Ballatori E. (1990). A randomized trial fo three cisplatin-containing regimens in advanced non-small-cell lung cancer (NSCLC): a study of the Umbrian Lung Cancer Group.. Cancer Chemother Pharmacol.

[PDF_00409] Crinó L., Darwish S., Corgna E., Meacci M. L., Di Costanzo F., Buzzi F., Fornari G., Santi F., Ballatori E., Luccioli L. (1988). Treatment of advanced non-small cell lung cancer (NSCLC): the "Umbria" cooperative study.. Semin Oncol.

[PDF_00416] Donnadieu N., Paesmans M., Sculier J. P. (1991). Chimiothérapie des cancers bronchiques non à petites cellules. Meta-analyse de la littérature en fonction de l'extension de la maladie.. Rev Mal Respir.

[PDF_00419] Einhorn L. H., Loehrer P. J., Williams S. D., Meyers S., Gabrys T., Nattan S. R., Woodburn R., Drasga R., Songer J., Fisher W. (1986). Random prospective study of vindesine versus vindesine plus high-dose cisplatin versus vindesine plus cisplatin plus mitomycin C in advanced non-small-cell lung cancer.. J Clin Oncol.

[PDF_00424] Fukuoka M., Masuda N., Furuse K., Negoro S., Takada M., Matsui K., Takifuji N., Kudoh S., Kawahara M., Ogawara M. (1991). A randomized trial in inoperable non-small-cell lung cancer: vindesine and cisplatin versus mitomycin, vindesine, and cisplatin versus etoposide and cisplatin alternating with vindesine and mitomycin.. J Clin Oncol.

[PDF_00429] Gandara D. R., Crowley J., Livingston R. B., Perez E. A., Taylor C. W., Weiss G., Neefe J. R., Hutchins L. F., Roach R. W., Grunberg S. M. (1993). Evaluation of cisplatin intensity in metastatic non-small-cell lung cancer: a phase III study of the Southwest Oncology Group.. J Clin Oncol.

[PDF_00433] Gridelli C., Airoma G., Incoronato P., Pepe R., Palazzolo G., Rossi A., Bianco A. R. (1992). Mitomycin C plus vindesine or cisplatin plus epirubicin in previously treated patients with symptomatic advanced non-small-cell lung cancer.. Cancer Chemother Pharmacol.

[PDF_00437] Israel L., Chahinian P., Depierre A. (1975). Response of 65 measurable epidermoid bronchogenic tumors of known spontaneous doubling time to four different chemotherapeutic regimens--strategic deductions.. Med Pediatr Oncol.

[PDF_00441] Kris M. G., Gralla R. J., Kelsen D. P., Casper E. S., Burke M. T., Fiore J. J., Cibas I. R., Heelan R. T. (1985). Trial of vindesine plus mitomycin in stage-3 non-small cell lung cancer. An active regimen for outpatient treatment.. Chest.

[PDF_00445] Luedke D. W., Einhorn L., Omura G. A., Sarma P. R., Bartolucci A. A., Birch R., Greco F. A. (1990). Randomized comparison of two combination regimens versus minimal chemotherapy in nonsmall-cell lung cancer: a Southeastern Cancer Study Group Trial.. J Clin Oncol.

[PDF_00449] Marino P., Pampallona S., Preatoni A., Cantoni A., Invernizzi F. (1994). Chemotherapy vs supportive care in advanced non-small-cell lung cancer. Results of a meta-analysis of the literature.. Chest.

[PDF_00452] Marino P., Preatoni A., Cantoni A., Buccheri G. (1995). Single-agent chemotherapy versus combination chemotherapy in advanced non-small cell lung cancer: a quality and meta-analysis study.. Lung Cancer.

[PDF_00455] Mascaux C., Paesmans M., Berghmans T., Branle F., Lafitte J. J., Lemaitre F., Meert A. P., Vermylen P., Sculier J. P., European Lung Cancer Working Party (ELCWP) (2000). A systematic review of the role of etoposide and cisplatin in the chemotherapy of small cell lung cancer with methodology assessment and meta-analysis.. Lung Cancer.

[PDF_00459] Masutani M., Akusawa H., Kadota A., Ohchi Y., Takahashi N., Tanigawa S., Koya Y., Horie T. (1996). A phase III randomized trial of cisplatin plus vindesine versus cisplatin plus vindesine plus mitomycin C versus cisplatin plus vindesine plus ifosfamide for advanced non-small-cell lung cancer.. Respirology.

[PDF_00464] Meert A. P., Berghmans T., Branle F., Lemaître F., Mascaux C., Rubesova E., Vermylen P., Paesmans M., Sculier J. P. (1999). Phase II and III studies with new drugs for non-small cell lung cancer: a systematic review of the literature with a methodology quality assessment.. Anticancer Res.

[PDF_00468] Mylonakis N., Tsavaris N., Bacoyiannis C., Karvounis N., Kakolyris S., Karabelis A., Beer M., Kosmidis P. (1992). A randomized prospective study of cisplatin and vinblastine versus cisplatin, vinblastine and mitomycin in advanced non-small cell lung cancer.. Ann Oncol.

[PDF_00472] Niell H. B., Griffin J. P., Hunter R. F., Meredith C. A., Somes G. (1989). Combination versus sequential single-agent chemotherapy in the treatment of patients with advanced non-small cell lung cancer.. Med Pediatr Oncol.

[PDF_00479] Ruckdeschel J. C., Mehta C. R., Salazar O. M., Cohen M., Vogl S., Koons L. S., Lerner H. (1981). Chemotherapy for inoperable, non-small cell bronchogenic carcinoma: EST 2575, generation II.. Cancer Treat Rep.

[PDF_00494] Samson M. K., Comis R. L., Baker L. H., Ginsberg S., Fraile R. J., Crooke S. T. (1978). Mitomycin C in advanced adenocarcinoma and large cell carcinoma of the lung.. Cancer Treat Rep.

[PDF_00497] Sculier J. P., Klastersky J., Dumont J. P., Vandermoten G., Rocmans P., Libert P., Ravez P., Becquart D., Mommen P., Dalesio O. (1986). Combination chemotherapy with mitomycin and vindesine in advanced non-small cell lung cancer: a pilot study by the Lung Cancer Working Party (Belgium).. Cancer Treat Rep.

[PDF_00502] Shinkai T., Eguchi K., Sasaki Y., Tamura T., Ohe Y., Kojima A., Oshita F., Saijo N. (1991). A randomised clinical trial of vindesine plus cisplatin versus mitomycin plus vindesine and cisplatin in advanced non-small cell lung cancer.. Eur J Cancer.

[PDF_00506] Veeder M. H., Jett J. R., Su J. Q., Mailliard J. A., Foley J. F., Dalton R. J., Etzell P. S., Marschke R. F., Kardinal C. G., Maksymiuk A. W. (1992). A phase III trial of mitomycin C alone versus mitomycin C, vinblastine, and cisplatin for metastatic squamous cell lung carcinoma.. Cancer.

[PDF_00514] Weick J. K., Crowley J., Natale R. B., Hom B. L., Rivkin S., Coltman C. A., Taylor S. A., Livingston R. B. (1991). A randomized trial of five cisplatin-containing treatments in patients with metastatic non-small-cell lung cancer: a Southwest Oncology Group study.. J Clin Oncol.

